# Smartphone Apps Using Photoplethysmography for Heart Rate Monitoring: Meta-Analysis

**DOI:** 10.2196/cardio.8802

**Published:** 2018-02-27

**Authors:** Benjamin De Ridder, Bart Van Rompaey, Jarl K Kampen, Steven Haine, Tinne Dilles

**Affiliations:** ^1^ University Hospital Ghent Ghent Belgium; ^2^ Department of Nursing and Midwifery Faculty of Medicine and Health Sciences University of Antwerp Antwerp Belgium; ^3^ Wageningen University Biometris Wageningen Netherlands; ^4^ StatUa Center for Statistics University of Antwerp Antwerp Belgium; ^5^ Department of Cardiology Antwerp University Hospital Edegem Belgium; ^6^ Department of Cardiology University of Antwerp Antwerp Belgium

**Keywords:** mobile applications, heart rate, photoplethysmography, electrocardiography, oximetry, meta-analysis

## Abstract

**Background:**

Smartphone ownership is rising at a stunning rate. Moreover, smartphones prove to be suitable for use in health care due to their availability, portability, user-friendliness, relatively low price, wireless connectivity, far-reaching computing capabilities, and comprehensive memory. To measure vital signs, smartphones are often connected to a mobile sensor or a medical device. However, by using the white light-emitting diode as light source and the phone camera as photodetector, a smartphone could be used to perform photoplethysmography (PPG), enabling the assessment of vital signs.

**Objective:**

The objective of this meta-analysis was to evaluate the available evidence on the use of smartphone apps to measure heart rate by performing PPG in comparison with a validated method.

**Methods:**

PubMed and ISI Web of Knowledge were searched for relevant studies published between January 1, 2009 and December 7, 2016. The reference lists of included studies were hand-searched to find additional eligible studies. Critical Appraisal Skills Programme (CASP) Diagnostic Test Study checklist and some extra items were used for quality assessment. A fixed effects model of the mean difference and a random effects model of Pearson correlation coefficient were applied to pool the outcomes of the studies.

**Results:**

In total, 14 studies were included. The pooled result showed no significant difference between heart rate measurements with a smartphone and a validated method (mean difference −0.32; 99% CI −1.24 to 0.60; *P*=.37). In adults, the Pearson correlation coefficient of the relation between heart rate measurement with a smartphone and a validated method was always ≥.90. In children, the results varied depending on measuring point and heart rate. The pooled result showed a strong correlation that was significant (correlation coefficient .951; 95% CI 0.906-0.975; *P*<.001). The reported limits of agreement showed good agreement between a smartphone and a validated method. There was a moderately strong significant negative correlation between the year of publication of the included studies and the mean difference (r=−.69; *P*<.001).

**Conclusions:**

Smartphone apps measuring heart rate by performing PPG appear to agree with a validated method in an adult population during resting sinus rhythm. In a pediatric population, the use of these apps is currently not validated.

## Introduction

### Background

Smartphone ownership rises year by year. Advanced economies still have the highest smartphone ownership rates. Smartphone ownership in countries with an emerging and developing economy, however, is rising at a stunning rate [1].

Due to their availability, portability, user-friendliness, relatively low price, wireless connectivity, far-reaching computing capabilities, and comprehensive memory, smartphones prove to be suitable for use in health care [2-4]. A wide offer of health and medical applications exist from diagnostic tools over professional education to apps supporting patients and health consumers [3,5]. In the field of cardiological literature, there has been a growing interest in mobile apps since 2003 [6].

### Measuring Vital Signs

Most of the studies focus on measuring vital signs using a smartphone. To this end, smartphones are mostly connected to a mobile sensor or medical device [6]. A majority of smartphones receive the information through built-in Bluetooth technology. They often process the information before transferring data to a server. At server level, the information can be further processed, organized, and analyzed to create a report for the user [2,4]. Hence, this type of monitoring requires several sensors or a separate device, which can be quite expensive [4].Another way to measure heart rate is by utilizing a pulse oximeter using photoplethysmography (PPG). In total, 2 key components are essential to create a PPG waveform: a light source to illuminate the subcutaneous tissue and a photodetector to detect the changes in light intensity [7]. Jonathan en Leahy demonstrated that a smartphone could be used to perform PPG. The white light-emitting diode can be used as light source and the phone camera as photodetector. The 2 components should be positioned next to each other for reflection mode PPG; in comparison, in transmission mode PPG, the photodetector is placed opposite to the light source [8].

The PPG waveform is influenced by many factors enabling the assessment of vital signs, for example, oxygen saturation, blood pressure, respiratory rate, and heart rate. Promising results show the ability to screen for pathologies related to peripheral vascular disease [7-9]. The purpose of this review was to analyze the available evidence on measuring heart rate by performing PPG using smartphones in comparison with a validated method.

## Methods

### Literature Search and Selection Criteria

We conducted a systematic literature search of PubMed and ISI Web of Knowledge from January 1, 2009 to December 7, 2016, with the following search key: (smartphone* OR phone* OR ((Applic* OR App*) AND (mobile OR electronic OR software)) OR PPG OR Photoplethysmograph* OR Rheograph*) AND (Electrocardiogr* OR ECG OR EKG or Oximet*) AND ((rate* AND (heart OR pulse)) OR tachycardia* OR beat* OR complex* OR arrhythmia* OR fibrillation*). Only papers in English, German, French, or Dutch were included. The reference lists of included studies were hand-searched to find additional eligible studies.

Studies were included if the measurement of heart rate was conducted with the photo camera of a smartphone by PPG; the measurements were made at a finger, toe, or earlobe; the measurements of the smartphone were compared with an electrocardiogram (ECG), a pulse oximeter, or another validated method to determine heart rate. Studies were excluded if the measurement was conducted with a mobile sensor or medical device connected to a smartphone; the paper did not have heart rate as one of the outcomes; no abstract or full text was available.

### Data Extraction and Outcome Measures

Data were extracted by the first author and reviewed by all authors.

Following are study and intervention characteristics extracted from the included studies: first author, study country, study year, sample size, baseline characteristics of participants, age of the participants (mean or range), type of smartphone used, control instrument, duration and conditions of the measurement, and primary outcome measures. The primary outcome measures were the mean difference between heart rate measured by a smartphone and a validated method, the correlation coefficient of the relation between heart rate measurements made by both methods, and the 95% limits of agreement derived from a Bland-Altman plot.

Overall, 1 author was contacted to receive missing data about the heart rate measurements; 2 authors were contacted because of a lack of clarity about the data; and 7 authors were contacted to get access to the full text of the paper; but 2 authors failed to respond to that last request.

### Study Quality

Study quality was appraised using the Critical Appraisal Skills Programme (CASP) Diagnostic Test Study checklist [10]. In addition, the included studies were evaluated by extra considerations described in the study of Hanneman [11]. The first was an appraisal tool developed for diagnostic studies. The checklist covered 3 sections: the validity of the results, the actual results, and the utility of the results. With the exception of the questions focusing on the actual results, the topics described were relevant for a method comparison study design. The 9 remaining questions were answered by “yes,” “can’t tell,” or “no.” One question was adapted so that “yes” always indicated a positive answer and “no” a negative answer. “Can’t tell” was answered when there was not enough information found in the study to answer the question. The checklist gave an indication of the quality per section and did not focus on a total score. The latter focused on specific considerations for a method comparison study design. The considerations were converted in 5 questions. These questions were also answered by “yes,” “can’t tell,” or “no.”

The quality assessment was performed by the first author and reviewed by the other authors.

### Statistical Methods

In total, 3 different statistics were described, and 2 of them were used for estimation of the pooled result. The first was the mean difference between heart rate measured by a smartphone and a validated method. In case of absence of a mean value and standard deviation in the original paper, it was calculated manually where possible on the basis of the original data.

The second was the Pearson correlation coefficient calculated from the relation between heart rate measured by a smartphone and a validated method. The *P* value was calculated manually out of the correlation coefficient and sample size if not described in the original paper.

The third were the 95% limits of agreement. They were derived from a Bland-Altman plot. Lower and upper limits were calculated starting from the mean difference by respectively subtracting and adding up the standard deviation of the mean difference between both methods, multiplied by a factor of 1.96. In 2 studies, they were calculated manually starting from the mean difference and the described limit of agreement.

The pooled result was estimated using a fixed- or random-effects model. Statistical heterogeneity was tested using the chi-squared test where a significant result indicated statistical heterogeneity. To quantify inconsistency, the I² of Higgins was used. In case of statistical heterogeneity, a random-effects model was used for pooling the results. Due to the small number of included studies, it was not possible to explore heterogeneity by subgroup analysis or meta-regression [12].

Pearson correlation was used to analyze the relation between different variables (publication year, mean heart rate, and sample size) and the mean difference. The scatter plots of these correlations were drawn.

Statistical analyses were performed using Review Manager Version 5.3 (The Cochrane collaboration, Copenhagen: Denmark: The Nordic Cochrane Centre, 2014), MedCalc 17.4 (MedCalc Software, Ostend: Belgium, 2017), and Microsoft Office Excel 2007 (Microsoft, 2007). Statistical significance level was set at 5%, except for mean difference where statistical significance level was set at 1%.

## Results

### Study Identification and Selection

**Figure 1:**
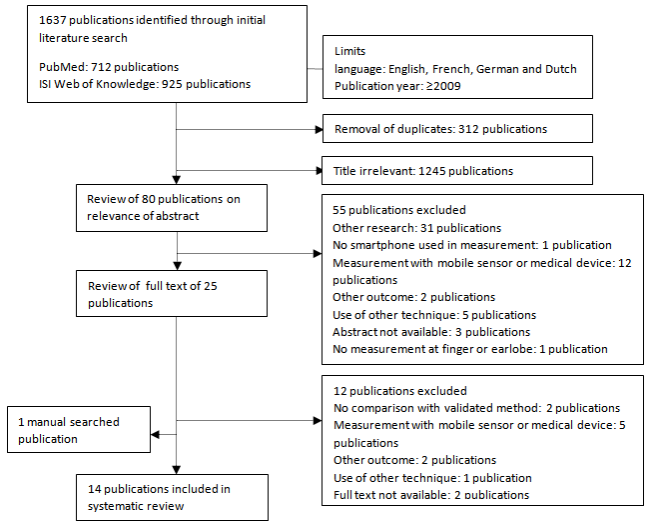
Search and selection strategy.

Figure 1 shows a diagram of the search and selection strategy. Initially, 1637 studies were found in 2 databases. First, 312 duplicates—identical studies found in both databases—were removed, followed by 1245 studies on the basis of an irrelevant title. The abstract of the remaining 80 studies was screened of which 55 were excluded for not fulfilling the selection criteria [4,13-66]. The 25 remaining studies were reviewed by reading the full text [8,67-90]. An additional 10 studies were excluded for not fulfilling the selection criteria [8,67,71,72,74,76,81,83,84,86]. For 2 studies, the full text could not be retrieved [69,77]. One paper was added after hand-searching the reference list of the included studies [91]. A total of 14 studies was used for this review and meta-analysis [68,70,73,75,78-80,82,85,87-91].

### Study Characteristics

Table 1 presents the characteristics of the included studies. In total, 5 studies reported findings on North American participants [68,73,85,89,90], 6 on Western European participants [70,78-80,87,91], and 3 on East Asian participants [75,82,88]. The oldest studies dated from 2010 and the most recent from 2016. Sample sizes varied from 1 to 68, with a median of 24. In total, 8 studies studied an adult population [70,73,78,80,82,85,87,91] and 2 an infant population [75,90], and 4 studies did not mention the age of the participants [68,79,88,89]. In 9 studies, the reference instrument was an ECG [68,70,75,78,79,82,85,89,90]; in 4 studies, a pulse oximeter [80,87,88,91]; and in 1 study, both [73]. The duration of the measurement varied between 10 s and 5 min. Of the selected studies, 2 did not mention the duration of the measurement [89,90]. A total of 5 studies tried to evoke variations in heart rate [68,73,78,82,91], 2 studies controlled the breathing of the participants during measurement [85,89], 1 paper made measurements in different lighting conditions [87], and 1 paper made measurements during different heart rhythms [90]. Overall, 8 studies studied another outcome besides heart rate, namely heart rate variability parameters [68,78,79], other vital parameters [85,89], and other outcomes [70,82,88].

### Study Quality

Table 2 presents the quality assessment of the included studies. The quality assessment questions are listed in Textbox 1. All studies had a clear study question and compared the measurements of the smartphone with an appropriate reference standard. Due to the type of test, it was not possible that the measurement of the reference standard influenced the measurement of the smartphone. Also, both methods did measure the same outcome simultaneously. Totally, 5 studies made a clear description of the disease status of the participants [70,73,80,82,90]. Just over half of the studies described the methods for performing the test in sufficient detail [68,70,73,75,78,80,82,85]. Half of the studies provided enough information about the participants to conclude that the results may be applicable to the population of interest [73,75,78,80,82,85,90]. All studies had the same relevant outcome and performed their measurements in a similar way. All but one [82] studies acknowledged that the sample size was small. In 6 studies, the authors made an effort to measure a wide range of the possible physiological values of heart rate [68,73,78,82,90,91]. Only 3 studies used a cutoff value for the clinical acceptable difference between the measurements made by the 2 methods [73,75,80].

### Primary Outcome: Heart Rate

**Figure 2:**
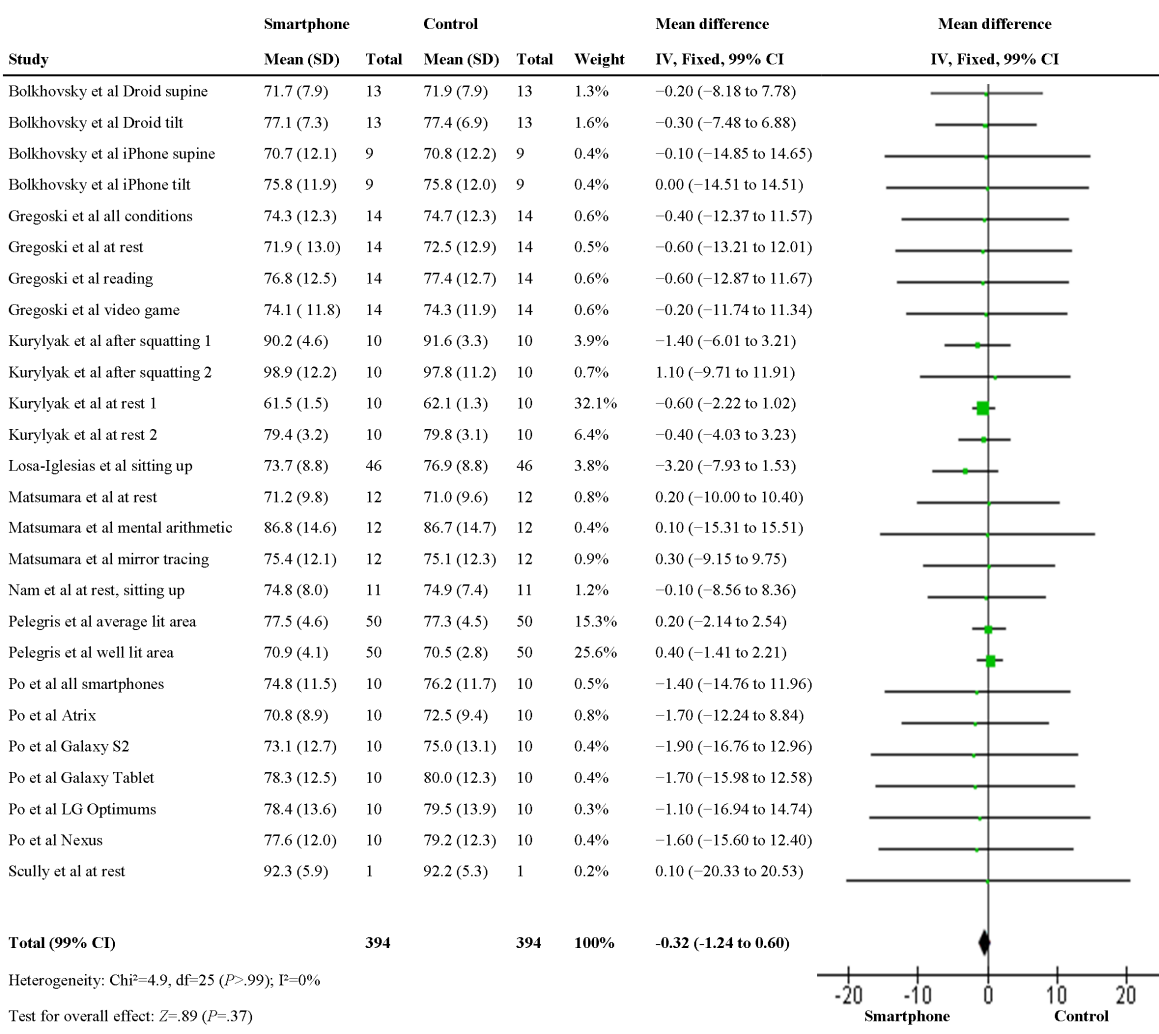
Forest plot for the meta-analysis of mean difference.

The mean difference between heart rate measured by a smartphone and a validated method was analyzed in a fixed-effects model (Figure 2). This statistic was reported in 7 studies [68,73,80,82,88,89,91]. For 2 studies, it was calculated manually out of the original data [85,87]. In 2 studies, the mean difference was consistently positive [82,89]; and in 5 studies, negative [73,80,85,87,88]. In 2 studies, the mean difference was negative, except for 1 condition where there was no difference [68] or the mean difference was positive [91]. The pooled estimate of the 9 included studies suggested that there is no difference between both methods (mean difference −0.32; 99% CI −1.24 to 0.60; *P*=.37). No statistical heterogeneity was observed among the studies (I²=0%; *P*>.99).

Table 3 shows the correlation coefficient of the relation between heart rate measurement with a smartphone and a validated reference method. This statistic was reported in 9 studies [68,70,73,75,78-80,82,90]. Previous research stated that the correlation between 2 methods that measure heart rate should be ≥.90 to be considered as valid [92]. In 7 studies, the correlation coefficient was always ≥.90 and the result was statistical significant [68,70,73,78-80,82]. The 2 studies that studied a pediatric population showed more variation in their results. In 1 , the correlation coefficients were remarkably lower during periods of tachycardia, namely .56 and −.43 [90] and not statistical significant for the latter. In 1 paper, the correlation coefficient was only ≥.90 in 2 of the 4 apps. In 1 of these 2 apps, this was just the case for measurements at the earlobe [75].

**Table 1:** Characteristics of included studies.

Author, year, and country	Sample size and age (range or mean [SD])	Smartphone	Control	Duration and conditions measurement	Outcome measure
Bolkhovsky et al, 2012, United States [68]	22 subjects, age not specified	Motorola Droid, iPhone 4S	ECG^a^	2 × 2 min: supine and sitting up in tilt position (iPhone 4S, n=9); 2 × 5 min: supine and sitting up in tilt position (Motorola Droid, n=13)	Heart rate, heart rate variability
Drijkoningen et al, 2014, Belgium [70]	28 adults with sinus rhythm during electrophysiological examination, age not specified	Samsung Galaxy S4	ECG	60 s	Heart rate, premature atrial ectopic beats identification
Gregoski et al, 2012, United States [73]	14 adults, 18-59 years	Motorola Droid	ECG, pulse oximeter	3 × 5 min: sitting, at rest, reading, and playing a video game	Heart rate
Ho et al, 2014, Taiwan [75]	40 children undergoing ECG monitoring, 3 days to 15 years	iPhone 4S	ECG	3 × 20 s at finger (or toe) and earlobe	Heart rate
Koenig et al, 2016, Germany [78]	68 adults (45 patients from a cardiologic outpatient ambulance and 23 healthy controls), 51.7 (18.83) years	iPhone 4S	ECG	5 min: at rest 2 min: after 3 min of physical exercise (only controls)	Heart rate, heart rate variability
Kurylyak et al, 2012, Italy [91]	10 adults, 26-60 years	HTC HD2, iPhone 4, Nokia 5800, Samsung Galaxy S i9000	Pulse oximeter	2 × 60 s (per smartphone): at rest and after 60 s squatting	Heart rate
Lagido et al, 2014, Portugal [79]	43 heart failure patients, age not specified	Sony Xperia S	ECG	At rest	Heart rate, heart rate variability
Losa-Iglesias et al, 2016, Spain [80]	46 healthy adults, 39.3 (7.35) years	Samsung Galaxy Note	Radial pulse, pulse oximeter	3 × 10-30 s: at rest (resting 10 min before measurements)	Heart rate
Matsumara et al, 2013, Japan [82]	12 students, 21-24 years	iPhone 4S	ECG	3 × 3 min: at rest (resting 7 min before measurement), during mental arithmetic, and during mirror tracing	Heart rate, normalize pulse volume
Nam et al, 2016, United States [85]	11 healthy nonsmoking adults, 20-40 years	HTC One M8	ECG	3 × 2 min: breathing at frequencies from 0.1 to 0.5 Hz at increments of 0.1 Hz, breathing at 1 Hz and spontaneous breathing	Heart rate and breathing rate
Pelegris et al, 2010, UK [87]	50 adults, 21-55 years	HTC Tattoo	Pulse oximeter	2 × 9 s: well-lit room and average lit room	Heart rate
Po et al, 2015, China [88]	10 subjects, age not specified	Samsung Galaxy Nexus, LG Optimus P920, Samsung Galaxy S2, Samsung Galaxy Tablet 7.0, Motorala Atrix	Pulse oximeter	1 × 20 s	Heart rate and root mean square distortion of heart rate
Scully et al, 2012, United States [89]	1 subject, age not specified	Motorola Droid	ECG	1 × ?: spontaneous breathing 3 × 2 min: breathing at 0.2, 0.3, and 0.4 Hz	Heart rate, respiration rate, oxygen saturation
Wackel et al, 2014, United States [90]	26 children undergoing an electrophysiology study under general anesthesia, 5-17 years	iPhone 5	ECG	2 × ?: during baseline heart rate (34 measurements in 17 children) 2 × ?: during sustained supraventricular tachycardia (38 measurements during 21 supraventricular tachycardia in 18 children)	Heart rate

^a^ECG: electrocardiogram.

**Table 2:** Study quality according to Critical Appraisal Skills Programme Diagnostic Test study checklist and extra considerations. Y indicates yes; N indicates no; and C indicates can’t tell.

Study	Validity of results						Utility of results				Extra considerations					
	Q1	Q2	Q3	Q4	Q5	Q6		Q9	Q10	Q11		E1	E2	E3	E4	E5
Bolkhovsky et al	Y	Y	Y	Y	N	Y		C	Y	Y		Y	Y	N	Y	N
Drijkoningen et al	Y	Y	Y	Y	Y	Y		C	Y	Y		Y	Y	N	N	N
Gregoski et al	Y	Y	Y	Y	Y	Y		Y	Y	Y		Y	Y	N	Y	Y
Ho et al	Y	Y	Y	Y	N	Y		Y	Y	Y		Y	Y	N	N	Y
Koenig et al	Y	Y	Y	Y	N	Y		Y	Y	Y		Y	Y	N	Y	N
Kurylyak et al	Y	Y	Y	Y	N	N		C	Y	Y		Y	Y	N	Y	N
Lagido et al	Y	Y	Y	Y	N	N		C	Y	Y		Y	Y	N	C	N
Losa-Iglesias et al	Y	Y	Y	Y	Y	Y		Y	Y	Y		Y	Y	N	N	Y
Matsumara et al	Y	Y	Y	Y	Y	Y		Y	Y	Y		Y	Y	Y	Y	N
Nam et al	Y	Y	Y	Y	N	Y		Y	Y	Y		Y	Y	N	N	N
Pelegris et al	Y	Y	Y	Y	N	N		C	Y	Y		Y	Y	N	N	N
Po et al	Y	Y	Y	Y	N	N		C	Y	Y		Y	Y	N	N	N
Scully et al	Y	Y	Y	Y	N	N		C	Y	Y		Y	Y	N	N	N
Wackel et al	Y	Y	Y	Y	Y	N		Y	Y	Y		Y	Y	N	Y	N

**Quality assessment questions.**

Critical Appraisal Skills Programme Diagnostic study checklist

- Validity of results
  - Was there a clear question for the study to address?
  - Was there a comparison with an appropriate reference standard?
  - Did all patients get the diagnostic test and reference standard?
  - Is there no possibility that the results of the test have been influenced by the results of the reference standard?
  - Is the disease status of the tested population clearly described?
  - Were the methods for performing the test described in sufficient detail?

- Utility of results
  - Can the results be applied to your patients/the population of interest?
  - Can the test be applied to your patient or population of interest?
  - Were all outcomes important to the individual or population considered?

Extra considerations

- Do both methods measure the same outcome?
- Do both methods measure the outcome simultaneous?
- Did the investigators motivate their choice for the sample size?
- Did the investigators test both methods in different conditions to simulate the possible physiological range of values?
- Did the investigators set up cutoff values for the clinical acceptable difference between both methods?

**Table 3:** Results for heart rate: Pearson correlation coefficient.

Study	Conditions (sample size)	*r* ^a^	*r* *≥*.90?	*P* value^b^
Bolkhovsky et al	iPhone supine (9)	>.99	Yes	<.001^c^
	iPhone tilt (9)	>.99	Yes	<.001^c^
	Droid supine (13)	.98	Yes	<.001^c^
	Droid tilt (13)	>.99	Yes	<.001^c^
Drijkoningen et al	Not specified (28)	.98	Yes	<.001
Gregoski et al	At rest (14)	.99	Yes	<.001^c^
	Reading (14)	.99	Yes	<.001^c^
	Video game (14)	.99	Yes	<.001
Ho et al	App A finger (40)	.81	No	<.001
	App A earlobe (40)	.91	Yes	<.001
	App B finger (40)	.75	No	<.001
	App B earlobe (40)	.76	No	<.001
	App C finger (40)	.27	No	.10
	App C earlobe (40)	.46	No	.003
	App D finger (40)	.90	Yes	<.001
	App D earlobe (40)	.98	Yes	<.001
Koenig et al	80 randomly chosen intervals at rest or after exercise (68)	>.99	Yes	<.001^c^
Lagido et al	At rest (43)	.94	Yes	<.001^c^
Losa-Iglesias et al	Sitting up (46)	.95	Yes	<.001
Matsumura et al	All conditions (12)	.99	Yes	<.001^c^
Wackel et al	App 1 sinus rhythm (17)	.99	Yes	<.001^c^
	App 1 tachycardia (10 succeeded attempts)	.56	No	.01^c^
	App 2 sinus rhythm (17)	.99	Yes	<.001^c^
	App 2 tachycardia (5 succeeded attempts)	−.43	No	.09^c^

^a^*r* value of Pearson correlation coefficient.

^b^*P* value calculated with Pearson correlation.

^c^Data based on own calculations.

**Figure 3:**
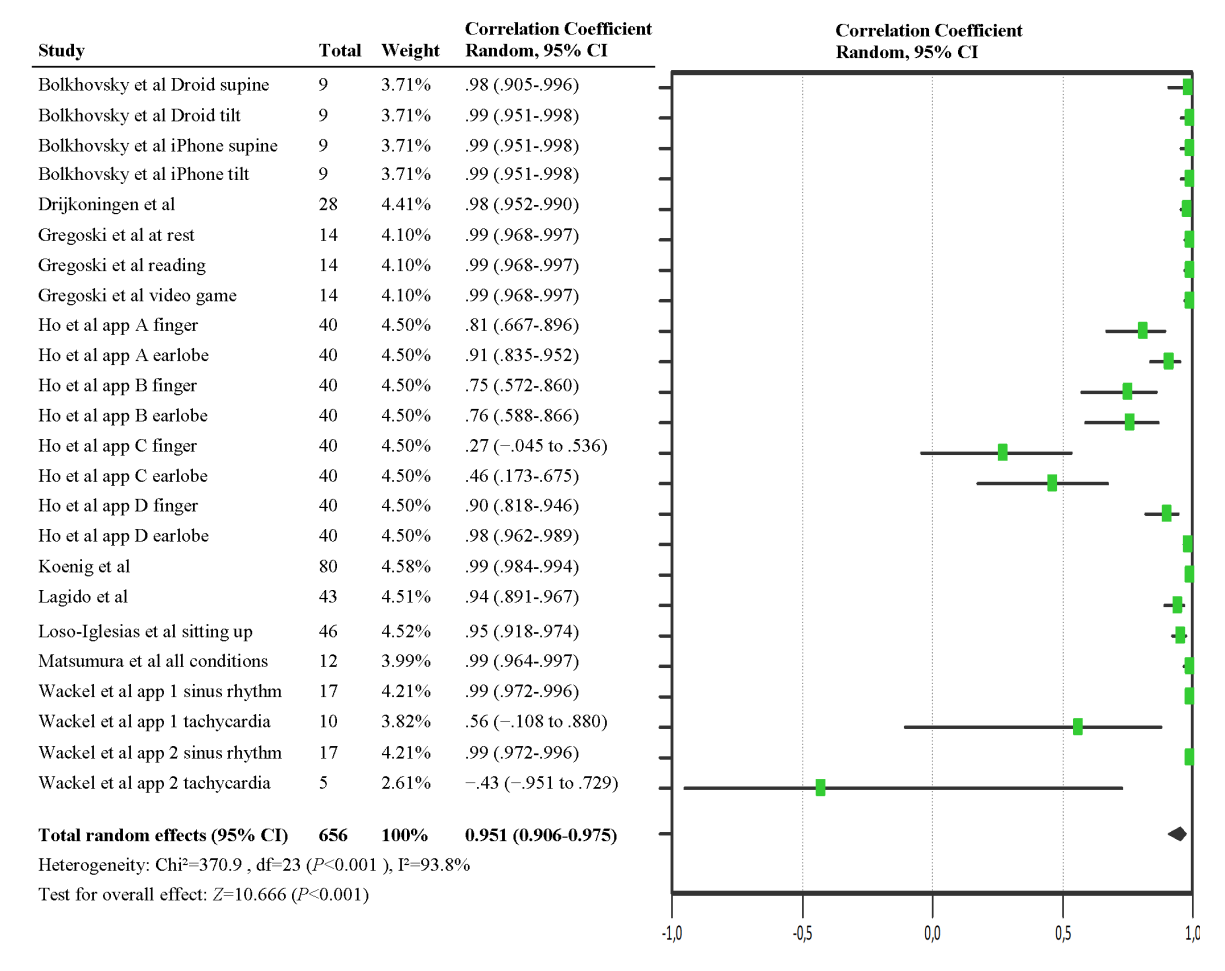
Forest plot for the meta-analysis of Pearson correlation coefficient.

The correlation between heart rate measurements made by a smartphone and a control instrument was analyzed in a random-effects model (Figure 3). The pooled correlation coefficient made the assumption that on average measurements made by a smartphone are highly correlated to those made by a control instrument (correlation coefficient .951; 95% CI 0.906-0.975; *P*<.001). Of note, statistical heterogeneity was high (I²=93.8%; *P*<.001), indicating variability across the studies.

Table 4 shows the 95% limits of agreement for the MD between measurements with a smartphone and a validated method. This statistic was reported in 4 studies [80,82,85,88]. For 2 studies, it was calculated manually [68,73]. In all studies, the limits of agreement did not exceed 10 beats per minute.

### Correlations With the Mean Difference

**Figure 4:**
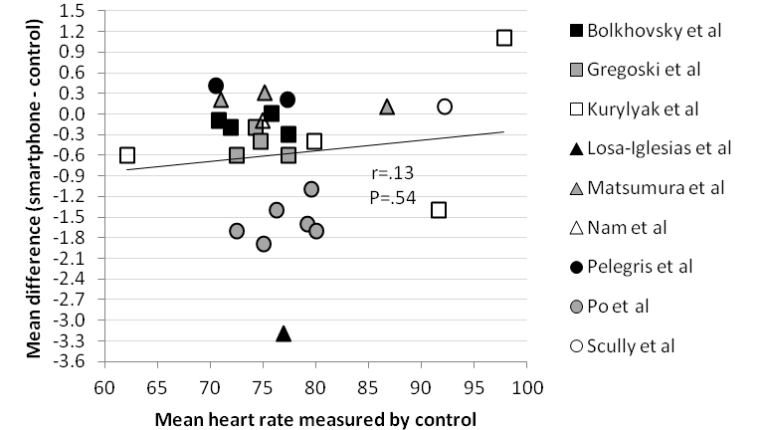
Scatter plot comparing correlation between mean heart rate measured by control and mean difference.

**Figure 5:**
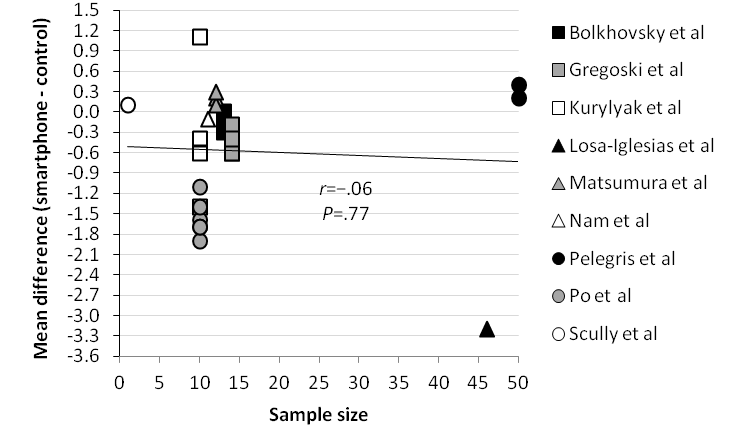
Scatter plot comparing correlation between sample size and mean difference.

**Figure 6:**
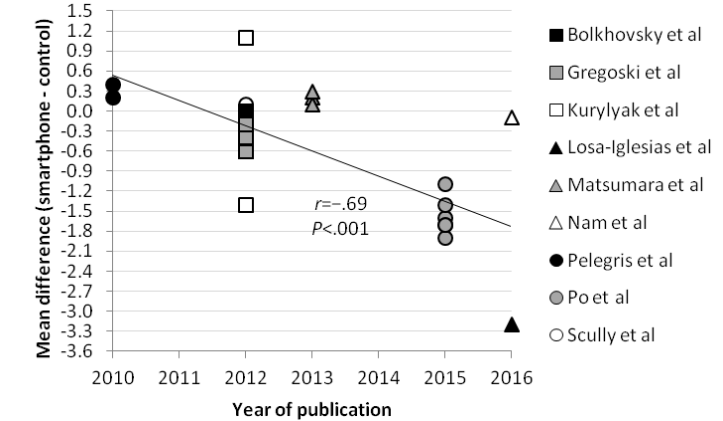
Scatter plot comparing correlation between year of publication and mean difference.

The correlation between the mean heart rate measured by a validated method, the sample size of the included studies, and the year of publication of the included studies and the mean difference was analyzed in Figures 4-6, respectively. Correlations between the mean difference and the mean heart rate measured by a validated instrument (*r*=.13) and sample size (*r*=−.06) were not significant. However, data showed a moderately strong correlation between the year of publication and the mean difference (*r*=−.69; *P*<.001).

**Table 4:** Results for heart rate: 95% limits of agreement.

Study	Conditions (sample size)	95% LOA^a^ (BPM^b^), control—smartphone
Bolkhovsky et al	iPhone supine (9)	−0.4 to 0.2^c^
	iPhone tilt (9)	−0.3 to 0.3^c^
	Droid supine (13)	−3.4 to 3.0^c^
	Droid tilt (13)	−1.7 to 1.1^c^
Gregoski et al	Video game (14)	−3.9 to 3.7^c^
Loso-Iglesias et al	Sitting up (46)	−8.5 to 2.0
Matsumura et al	All conditions (12)	−1.0 to 1.4
Nam et al	At rest, sitting up (11)	−5.6 to 5.5
Pot et al	Average all smartphones (10)	−4.1 to 1.2

^a^LOA: limits of agreement.

^b^BPM: beats per minute.

^c^Data based on own calculations.

## Discussion

### Principal Findings

The meta-analysis of the mean difference showed no statistical difference between the measurement of heart rate by a smartphone and a validated method (mean difference −0.32; 99% CI −1.24 to 0.60; *P*=.37). The pooled correlation coefficient between heart rate measurement by a smartphone and a validated method was more than .90 and statistically significant (correlation coefficient .951; 95% CI 0.906-0.975; *P*<.001). Reported 95% limits of agreement had a narrow range and therefore showed good agreement between a smartphone and a validated method. These results suggest that a smartphone app deriving heart rate from a PPG signal could be used as an alternative for already validated methods such as an ECG or pulse oximeter in an adult population in resting sinus rhythm. However, the significant negative correlation between the year of publication of the included studies and the mean difference (*r*=−.69; *P*<.001) suggests that smartphone technology for measuring heart rate did not improve over time. There was no significant correlation between the mean difference and the mean heart rate measured by a validated method (*r*=.13; *P*=.54) or the sample size of the included studies (*r*=−.06; *P*=.77), which suggests that smartphone results are consistent for heart rate measurements between 60 and 100 beats per minute.

### Considerations

First, the results of the studies in a pediatric population showed that it is not advisable yet to use these apps in children. A possible cause is that because of the smaller size of children’s fingertips, the pulsatile flow may be less consistently detected. The use of the earlobe as a measuring point may present a possible solution. Children may also have difficulties in containing the appropriate pressure on the camera lens and keeping their finger motionless to make a good measurement [73,75,90].

A second issue is heart rate measurement during periods of arrhythmia [4]. The low correlation between measurements with a smartphone and a validated method during periods of supraventricular tachycardia in children suggests that current apps do not give adequate results during periods of extremely high heart rates [90]. Moreover, the smartphone apps in the studies used PPG, calculating the heart rate on basis of the pulse rate. Hence, the results may not be accurate enough during periods of arrhythmia with variations in pulse rate and amplitude due to heart rhythm irregularities [4,82]. A solution is to improve sensitivity and specificity of the apps for deviant heart rhythms depending on the purpose of the apps [62].

Third, previous research stated that heart rate measurement can be susceptible to environmental or human factors such as ambient light, motion [4,93], or skin color [7]. In total, 3 studies reported about lighting conditions [87,88,91]. In these studies, ambient light did not seem to have an influence, but it should still be taken into account. On the basis of this review, it is not possible to say something about the influence of motion, as none of the included studies tested whether accurate pulse rate is measurable by the smartphone apps during exercise. However, several studies do mention this limitation in their discussion. Wearable devices using PPG possibly provide better results during exercise [94]. Only 1 paper mentioned to have included participants with a variety of skin colors but did not make a comparison between different skin types [73]. Hereby, we cannot come to a conclusion about the topic in this review. When using PPG to measure heart rate, it should be taken into account to use a proper light wavelength that gives equal results for people with different skin types [95].

Fourth, it was remarkable that in the included studies the mean difference became more and more negative over time. A plausible explanation is that every paper focuses on (a) certain type(s) of smartphone model(s) or app(s). Consequently, the results cannot be automatically projected to other smartphones and apps [4]. The use of certain smartphones or apps could lead to better results.

### Strengths and Limitations

First of all, to the best of our knowledge, this was the first systematic review and meta-analysis evaluating smartphone apps using PPG to measure heart rate. A comprehensive search strategy was used, including every paper investigating smartphone apps deriving heart rate measurement from a PPG signal. At last, there was a focus on different statistics for assessing agreement between methods.

Nevertheless, there were some limitations of the included studies. First, the methodological quality was often low, reflected by the fact that only 3 studies scored 12 or more out of 14 on the quality assessment questions [73,80,82].

Second, most of the mean heart rates that were reported lay between 70 and 80 beats per minute. As a result, it was not possible to investigate whether smartphones could be used to measure the higher physiological ranges of heart rate.

Third, only 8 of the included studies [68,70,73,78,80,82,85,88] used the most appropriate method to determine agreement between the 2 methods, the Bland-Altman plot [96,97]. Of these studies, only 2 mentioned a conclusion of the results, which were in line with the findings of the review [70,78]. A consideration about this method is that it is not easy to determine good agreement [96]. In the literature, no description was found of the maximum heart rate deviation to be clinical relevant. A deviation of under 10 beats per minute has no important clinical implications but does indicate small alterations when repeating the measures. The other methods can support the findings but have their limitations. A Pearson correlation gives information about the relation between methods, but a high correlation does not necessarily mean that the 2 methods agree [97,98]. When using a mean difference, poor agreement can be hidden by looking at the mean difference, without exploring the individual values (eg, an overestimation of high heart rates in combination with an underestimation of low heart rates will also give a mean difference of 0) [97].

A fourth and last limitation is a high statistical heterogeneity between studies on the level of correlation coefficients. This is likely attributable to clinical heterogeneity caused by differences in patient characteristics (eg, adults vs children), the conditions in which the heart rates were measured (eg, at sinus rhythm vs during a period of tachycardia), and which smartphone or app was used [12].

All these factors may influence the generalizability of the results.

In addition, there were some limitations specific to the review. The data were extracted by the first author only; however, they were thoroughly reviewed by the other authors, of which one is specialized in cardiology. In addition, 2 studies were excluded because the full text could not be retrieved [69,77]; the results described in the abstracts of those studies agreed with the pooled results, so their exclusion would probably have a minimal effect.

### Conclusions

This meta-analysis suggests that heart rate measured by smartphone apps performing PPG agrees with a validated method in an adult population in resting sinus rhythm, provided that during measurement the measuring point was kept still and that appropriate pressure was maintained. In a pediatric population, the use of these apps can currently not be supported, especially not during periods of tachycardia. Future research with a larger and more diverse study population should be conducted. The technology should also be tested in more varied clinical situations evoking variations in normal heart rate and during arrhythmias.

## Abbreviations

CASP: Critical Appraisal Skills Programme
ECG: electrocardiogram
PPG: photoplethysmography
